# Herb Formula ZhenRongDan Balances Sex Hormones, Modulates Organ Atrophy, and Restores ER*α* and ER*β* Expressions in Ovariectomized Rats

**DOI:** 10.1155/2018/5896398

**Published:** 2018-06-13

**Authors:** Xuan Zhang, Qian Chen, Bo Chen, Fangqin Wang, Xiao-Hong Chen

**Affiliations:** ^1^Department of Pharmacology, Third Military Medical University (Army Medical University), Chongqing 400038, China; ^2^Biomedical Analysis Center, Third Military Medical University (Army Medical University), Chongqing 400038, China

## Abstract

Herb mixtures are widely used for treatment of the menopausal syndrome long before the hormonal therapy. However, there is insufficient data for herb remedies in treating menopausal syndromes. Here we aim to investigate the effect of ZhenRongDan (ZRD) in balancing female hormones, regulating expression of estrogen receptors (ERs), and preventing organ atrophy in menopausal rats. Rats that underwent bilateral ovariectomy were used in the experiments; the effects of ZRD on serum follicle-stimulating hormone (FSH), luteinizing hormone (LH), prolactin (PRL), and estradiol (E2) levels were observed. Histology of vagina and ERs expression in vagina, uterus, and adrenal gland were also examined. ELISAs were used to analyze the changes of FSH, LH, PRL, and E2 in serum, and the morphological changes of the cervical epithelium cells were observed by Hematoxylin & Eosin (H&E) staining. Immunohistochemistry and western blot were applied to detect estrogen receptors subtypes alpha (ER*α*) and beta (ER*β*) expression in vagina, uterus, and adrenal gland. We found that ZRD could significantly reduce the weight of the adrenal gland and increase the weight of the uterus. It could decrease the release of FSH and LH as well as increasing E2 and PRL levels. Furthermore, ZRD could improve the number of cervical vaginal epithelial cells and increase the thickness of the vaginal wall. And the altered expressions of ER*α* and ER*β* are also restored by ZRD. ZRD could obviously relieve the endocrine disorders, modulate organ atrophy, and restore ER*α* and ER*β* expression in the ovariectomized rat model.

## 1. Introduction

Menopausal syndrome refers to a series of symptoms of women with the disorder of autonomic nervous system caused by the fluctuation of estrogen level [1, 2]. The main pathogenesis is the decrease of estrogen secretion caused by the decline of ovarian function and the dysfunction of hypothalamic-pituitary-ovarian axis [3]. Since the long-term use of estrogen could increase the incidence of breast cancer or endometrial cancer [4, 5], hormone replacement therapy was restricted for use [6]. Traditional Chinese medicine (TCM) is an important aspect of alternative therapy for menopausal syndrome [7, 8]. Though many herbs are effective in the treatment of menopausal syndrome with different mechanisms [9, 10], the Chinese herb mixture has been used to treat menopausal syndrome for thousands of years [11]. According to the TCM theory, menopausal syndrome is caused by kidney-liver weakness based on yin-yang imbalance and organ disharmony [12]. However, the gap between the traditional complementary alternative medicine and the conventional main stream western medicine needs to be filled by more experimental and clinical research [13]. Thus it would be necessary to evaluate these Chinese herbs and make them working better in menopausal patients. 

Chinese herb mixture often acts like a super “cocktail”. Hence, an acknowledged evaluation system and appropriate parameters are needed to objectively evaluate the effect of these herbs [14]. Here we introduce a herb mixture named ZRD developed from the classic herb formula Siwu decoction. Siwu decoction usually contains the following four herbs: Radix rehmanniae, Radix Paeoniae alba, Angelica sinensis, and Ligusticum chuanxiong Hort. Siwu decoction is a gold formula widely used for treating gynecological diseases [15]. It has been applied since the late Tang dynasty and its bioactive constituents have been recently elucidated [16]. ZRD is based on Siwu decoction and consists of 17 kinds of medicinal herbs, which has been approved by China Food & Drug Administration as a traditional formula in treating menopausal syndromes [17]. These herb mixtures are composed to tonify the liver and kidney, nourish deficient in organs, and finally balance yin and yang. However, its working mechanism has not yet been elucidated. 

Previously, our group reported the effect of ZRD on behavior in menopausal mice [18], exhibiting strong potential in alleviating menopausal symptoms. Thus we explore the mechanism of ZRD further. Since they are central in physiological and pathological process [19], we evaluate the effect of ZRD in regulating sex hormone levels of menopausal rats. Meanwhile, its impact on the expression of ER*α* and ER*β* is addressed as estrogen receptors are vital for the development and function of the female organ [20, 21]. The influence of ZRD on adrenal gland and climacteric rats' uterine weight is also elucidated.

## 2. Materials and Methods

### 2.1. Herb Contents

ZRD is a herb mixture of TCM consisting of 17 herbs. The details of ZRD are listed in Table 1. Basic information such as Chinese name, Latin name, English name, and the part used in the mixture is covered.

### 2.2. Animals and Treatment

All animal experiments comply with the ARRIVE guidelines and were carried out in accordance with the UK Animals (Scientific Procedures) Act, 1986, and associated guidelines, EU Directive 2010/63/EU for animal experiments. The protocol was approved by the Laboratory Animal Welfare and Ethics Committee of the Third Military Medical University (SYSK-PLA-20120013, 2015.11.17). All efforts were made to minimize animal suffering and to reduce the number of animals used. Three-month old female Sprague-Dawley rats with the weights 180~220 g were obtained from the Experimental Animal Center of Third Military Medical University, China. A total of 82 specific pathogen free Sprague-Dawley female rats were used. All rats underwent bilateral ovariectomy as previously described [22] except the sham group. All OVX rats were checked by daily vaginal epithelial cell smear analysis, in which 5 consecutive days of leukocytes were indicative of constant diestrus and successful ovariectomy. After acclimatization for a week, the rats were randomly divided into 5 groups: rats that underwent sham-operation (sham); OVX without treatment (OVX); OVX rats treated with ZRD 3.6 g/kg, 0.01 ml/g (ZRD); OVX rats treated with Tai tai-le (one of the most popular commercial decoctions in China) 7.79 g/kg, 0.01 ml/g (TTL); and OVX rats tested with estradiol valerate 800 ug/kg, 0.01 ml/g (EV). Untreated control OVX rats and sham-operated rats received distilled water only. TTL and EV were used as the positive control. Dose calculations followed guidelines correlating dose equivalents between humans and laboratory animals, on the basis of ratios of body surface area. In our previous work, we found that ZRD exerts the best effect at the middle dose (5.2 g/kg) in mice, in contrast to the low dose (1.73 g/kg) and high dose (15.2 g/kg) [18]. Thus, rats in ZRD group were treated at the dose of 3.6 g/kg, which is equivalent to the middle dose in mice. All the above groups toke intragastric drug at approximately 16:00 ~ 18:00 pm every day for one month. All animals were maintained on the 12 h light/dark cycle under constant temperature (24 ± 2°C) and humidity (55 ± 5%), being allowed free access to food and water.

### 2.3. Analysis of Tissue and Serum

Animals were executed by cervical dislocation after one-month treatment. In sham group, all rats were in the estrus stage. Blood was collected from heart for analysis of serum E2, FSH, PRL, and LH levels by enzyme-linked immunosorbent assay (ELISA) (USCN, USA). The sensitivities of the four ELISAs were 1.0 pg/mL, 1.0 ng/mL, 1.0 ng/mL, and 1.0 pg/mL, respectively. And the antibody did not cross-react with other estrogen-like substances. The uterus and adrenal gland were removed and weighed. The left horns of the uterus and the mammary gland were stored at – 80°C and the rest of vagina was fixed with 4% polyoxymethylene for 24 h. The samples were embedded in paraffin and prepared for cross sections. 4 mm thick sections were cut, mounted, and stained with H&E for microscopy. The observation of the vaginal epithelial thickness was performed on a selected single slide in each animal. With randomly selected six areas from each tissue section, the average thickening value of vaginal epithelial cells was measured by the Image-Pro Plus 6.0 system image analysis software.

### 2.4. Immunohistochemistry

Immunohistochemistry procedure followed previously reported methods [23]. Briefly, 4 mm thick tissue section of vagina was mounted on polylysine-coated slides. The paraffin sections were dewaxed and incubated for 10 min with 3% hydrogen peroxide. Each section was incubated with blocking serum (Vectastain, ABC Kit) at room temperature for 30 min and then overnight at 4°C with rabbit anti-ER*α* polyclonal antibody (Abcam Biotechnology, UK) and rabbit anti-ER*β* polyclonal antibody (Abcam Biotechnology, UK), respectively. Sections incubated in phosphate-buffered saline without antibody served as negative controls. Positive control experiments for ER*α* and ER*β* were performed in adult Sprague-Dawley female rat's uterus. After incubation with biotinylated secondary antibody, sections were incubated with avidin-biotin complex reagent containing horseradish peroxidase for 30 min. The sections were then stained with 3,3'-diaminobenzidine (Sigma, USA). The Image-Pro Plus 6.0 System image analysis software was used for quantitative analysis.

### 2.5. Western Blot

Uterus was resuspended in lysis buffer (50 mM Tris, pH 8.0, 150 mM NaCl, 5 m MEDTA, 0.1% sodium dodecyl sulfate, 0.5% NP-40) containing 10 mM phenylmethylsulfonyl fluoride and 2 mg/mL aprotinin. The protein was obtained to detect the levels of ER*α* and ER*β* in target tissue by western blot. Western blot protocol and semiquantitative analysis were carried out as described in [24]. The rabbit anti-ER*α* polyclonal antibody (Abcam Biotechnology, UK) or rabbit anti-ER*β* polyclonal antibody (Abcam Biotechnology, UK) was used. The experiment was done in triplicate. The relative quantity of each antibody was measured by Quantity One software. The density ratio of protein to GAPDH (Sangon Biotech, Shanghai, China) was calculated from the band density.

### 2.6. Statistical Analysis

The SPSS version 13.0 for Windows software (SPSS Inc., Chicago, USA) was used for statistical analysis. All data were expressed as the mean standard deviation and were analyzed by one-way analysis of variance (ANOVA). Differences were considered statistically significant when the *p*-value was less than 0.05.

## 3. Results

### 3.1. Effect of ZRD on Uterine and Adrenal Gland Weights

**Figure 1:**
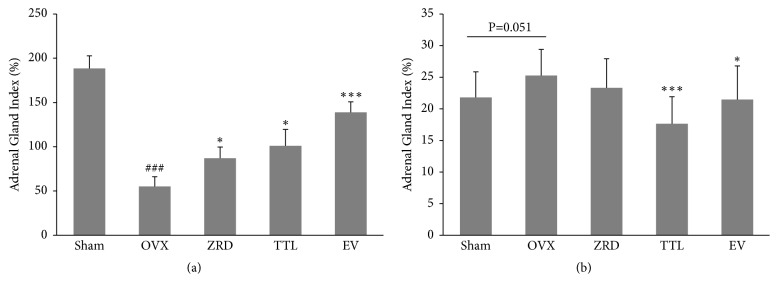
Effect of ZRD on uterine and adrenal gland weight. (a) Uterus index in sham, OVX, ZRD, TTL, and EV group. (b) Adrenal gland index in sham, OVX, ZRD, TTL, and EV group. Data were the mean standard deviation (SD) of samples. (^###^*p* < 0.001, compared with the sham group; ^*∗*^*p* < 0.05, compared with the OVX group; ^*∗∗∗*^*p* < 0.001, compared with the OVX group; one-way ANOVA.)

Uterus and adrenal gland weights largely reflect the alteration of female function [25]. To this end, we measured the uterine and adrenal glands weight in each group. The weights of uterus and adrenal gland were measured at the end of the one-month treatment period. As shown in Figure 1, the mean uterine index of OVX rats was significantly reduced (*p* < 0.001) compared with sham group. In contrast, the mean adrenal gland index was increased slightly. Further, the mean uterine indexes of rats in the ZRD group, TTL group, and EV group were increased compared to that of the rats in the OVX group, with the mean uterine index of EV group being statistically significant (*p* < 0.001). And the adrenal indexes in both ZRD and EV groups were slightly decreased compared with the OVX rats. As expected, TTL group's adrenal index was decreased significantly (*p* < 0.001).

### 3.2. ZRD Regulates the Levels of FSH, LH, PRL, and E2 in Serum

**Figure 2:**
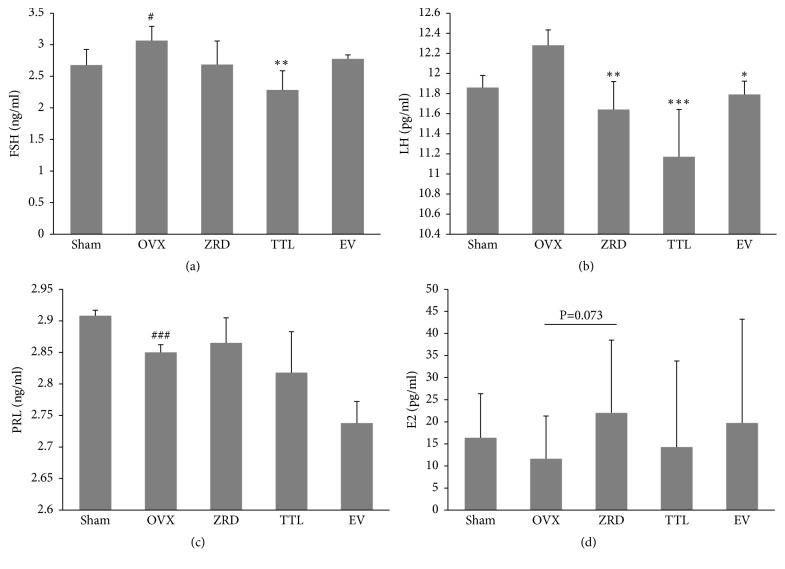
Effect of ZRD on serum FSH, LH, PRL, and E2 level in rats. (a) Serum FSH level in sham, OVX, ZRD, TTL, and EV groups. (b) Serum LH level in sham, OVX, ZRD, TTL, and EV groups. (c) Serum PRL level in sham, OVX, ZRD, TTL, and EV groups. (d) Serum E2 level in sham, OVX, ZRD, TTL, and EV groups. Data are the mean standard deviation (SD) of samples. (^#^*p* < 0.05, compared with the sham group; ^###^*p* < 0.001, compared with the sham group; ^*∗*^*p* < 0.05, compared with the OVX group; ^*∗∗*^*p* < 0.01, compared with the OVX group; ^*∗∗∗*^*p* < 0.001, compared with the OVX group; one-way ANOVA.)

Hormones such as FSH, LH, PRL, and E2 play critical role in regulating functions in female [26]. The reproductive endocrinology undergoes menopausal transition in the hormone patterns during menopause [27]. To elucidate the sex hormone changes in each group, we then test the levels of each serotonin in the rats' serum. As shown in Figure 2, ovariectomy resulted in lowered PRL and E2 level, but higher LH level and significantly elevated FSH (*p* < 0.05) levels in serum compared with sham group. When those OVX rats were given ZRD, TTL, and EV for one month, hormones in serum were regulated towards the normal status. Compared with the OVX rats, serum LH levels in ZRD and EV groups were decreased (*p* < 0.01, *p* < 0.05). Further, serum FSH and LH levels in TTL group were both significantly decreased (*p* < 0.01, *p* < 0.001). Similarly, the levels of PRL in serum of ZRD, TTL, and EV groups were slightly increased compared with the OVX rats. And serum E2 levels of rats in ZRD group were slightly increased compared with that of OVX rats (*p* = 0.073).

### 3.3. Effect of ZRD on the Histology of Vagina

**Figure 3:**
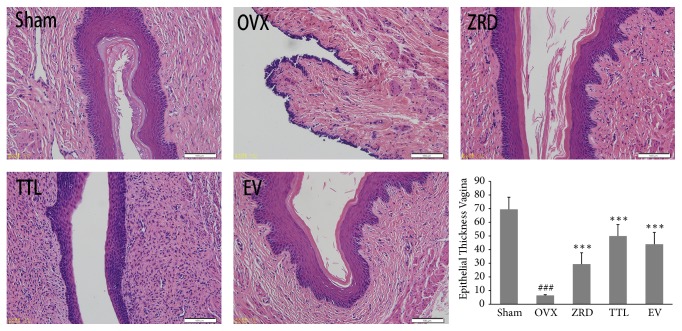
Effect of ZRD on the histology of vagina in rats. Representative pictures of vagina histology in sham, OVX, ZRD, TTL, and EV groups are shown. All pictures are stained with H&E and examined under ×20 magnification. Quantitative analyses of epithelial thickness in each group are also shown. (^###^*p* < 0.001, compared with the sham group; ^*∗∗∗*^*p* < 0.001, compared with the OVX group; one-way ANOVA.)

Vagina function undergoes sensible variation during menopausal syndrome [28] and vaginal atrophy is a common symptom of postmenopausal estrogen deficiency [29]. To prove that ZRD could benefit the vagina function, we examined the morphology of vagina by immunohistology. OVX control group showed atrophic vaginal epitheliums composed of a few layers of flattened cells. However, one-month administration of ZRD at all doses caused a complete reversal of the vaginal atrophy. This effect was accompanied by hyperplasia and hypertrophy of vaginal epithelium. As shown in Figure 3, the vaginal mucosa overlying in sham group shows thicker stratified squamous epithelium. Compared to sham group, we observe vaginal atrophy and vaginal epithelial thinning in OVX model group, with low degree of diversification. However, the vaginal epithelium was thickening and vaginal epithelial cells were all increased in ZRD, TTL, and EV groups compared with the OVX model group. Furthermore, the vaginal walls were significantly thicker than that of OVX rats (*p* < 0.001).

### 3.4. Expression of ER*α* and ER*β* in Vagina

**Figure 4:**
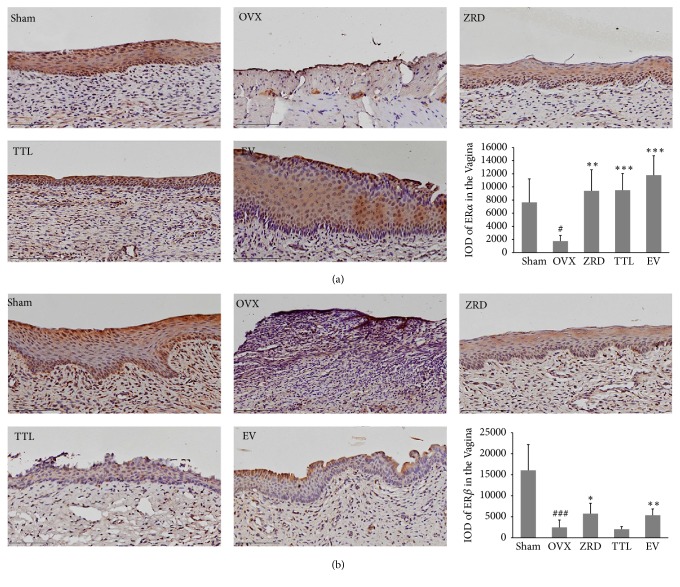
Effect of ZRD on the expressions of ER*α* and ER*β* in the vagina. (a) ER*α* expressions were assessed by quantitative immunohistochemistry. Representative photomicrographs taken at 200× magnification of vagina sections from each treatment group are shown. Quantitative analyses of ER*α* IOD are also shown. (b) ER*β* expressions were assessed by quantitative immunohistochemistry. Representative photomicrographs taken at 200× magnification of vagina sections from each treatment group are shown. Quantitative analyses of ER*β* IOD are also shown. Data are the mean standard deviation (SD) of samples. (^#^*p* < 0.05, ^###^*p* < 0.001, compared with the sham group; ^*∗*^*p* < 0.05, ^*∗∗*^*p* < 0.01, ^*∗∗∗*^*p* < 0.001, compared with the OVX group; one-way ANOVA.)

ERs were localized mainly in the superficial layer of the stratified squamous epithelium, blood vessel walls, and muscle fibers of the vagina [30]. The overall proliferative response to E2 is the result of a balance between ER*α* and ER*β* signaling [31]. To assess the alteration of ERs in response to different treatment, we then detect ER*α* and ER*β* levels in vagina by immunohistochemistry. As shown in Figure 4, representative sections of the expressions of ER*α* and ER*β* in the vagina from each group and quantitative analysis were shown. Importantly, the expressions of ER*α* and ER*β* in the vagina epithelium of OVX rats were both significantly decreased compared to the sham group (*p* < 0.05, *p* < 0.001). As expected, the vagina epithelium expressions of ER*α* in ZRD, TTL, and EV groups were significantly higher compared with that of the OVX group (*p* < 0.01, *p* < 0.001, and *p* < 0.001, respectively). In addition, the vagina epithelial expression of ER*β* in ZRD group was increased compared with that of the OVX model group (*p* < 0.05), consistent with that of EV group (*p* < 0.01). We also performed the negative and positive control experiment for ER*α* and ER*β* (data not shown).

### 3.5. Protein Levels of ER*α* and ER*β* in Uterus and Adrenal Gland

**Figure 5:**
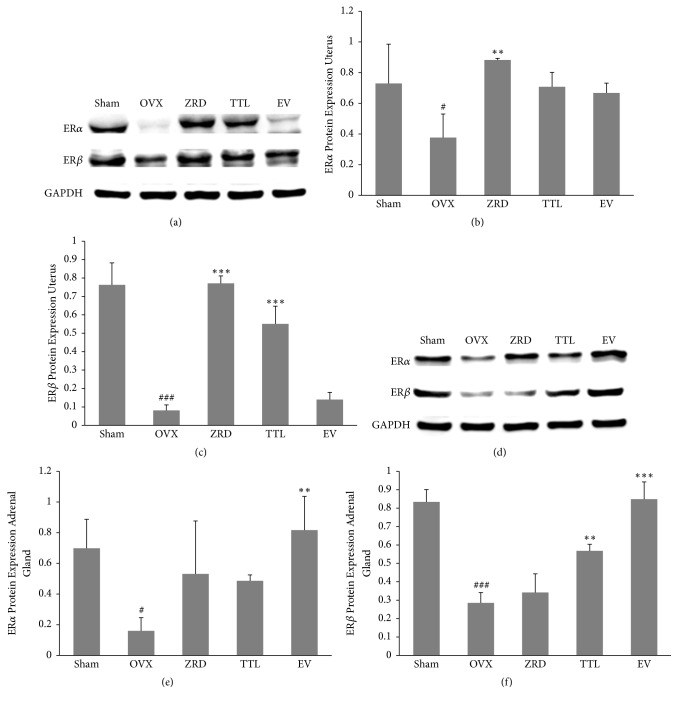
Effect of ZRD on the expressions of ER*α* and ER*β* protein in uterus and adrenal gland. (a) Representative blots of ER*α* and ER*β* protein in uterus. (b) Quantitative analyses of ER*α* expression in A. (c) Quantitative analyses of ER*β* expression in A. (d) Representative blots of ER*α* and ER*β* protein in adrenal gland. (b) Quantitative analyses of ER*α* expression in D. (c) Quantitative analyses of ER*β* expression in D. Results from each antibody were normalized against the level of GAPDH signal detected. Each experiment was repeated for three times. (^#^*P* < 0.05, ^###^*p* < 0.001, compared with the sham group; ^*∗∗*^*p* < 0.01, ^*∗∗∗*^*p* < 0.001, compared with the OVX group; one-way ANOVA.)

Further evidence for the interaction of the ZRD with the ER system was sought by determining the effects on ERs expressions in target tissues by western blot. To this end, the expressions of ERs in the uterus and adrenal gland were shown in Figure 5. Compared with the sham group, the expressions of ER*α* and ER*β* were significantly decreased in the uterus and adrenal gland of OVX rats. Significantly, the expression of ER*α* in the uterus and the expression of ER*β* in the adrenal gland were most decreased (*p* < 0.001) (Figures 5(b) and 5(f)). Moreover, the expressions of ER*α* and ER*β* in ZRD group were both increased in the uterus and adrenal glands, with the expression of ER*α* (*p* < 0.001) and ER*β* (*p* < 0.01) in the uterus significantly increased. And the expressions of ER*α* and ER*β* in the adrenal glands were increased, but there was no significant difference.

## 4. Discussion

Alternative medicine especially traditional oriental medicine called much attention in battle with certain ailments [32]. Interestingly, herbs are usually very useful in treating disorders that lack obvious pathological alterations such as menopausal syndrome [33, 34]. Previously, natural product such as soybean extract is reported to proliferate the vagina of adult rats [35] and could lower the cancer risk comparing to estrogen treatment [36]. Moreover, medicinal plants such as sage herb and lemon balm were found to play an imperative role in the treatment of acute menopausal syndrome [9]. However, the major concerns about the application of herb mixture are the safety and its unclear working mechanism. Thus, to elucidate the working mechanism would popularize herbs in future medication. Here our study provides new evidence suggesting that ZRD might relieve menopausal disorders in rats.

Importantly, ZRD showed strong potential in relieving menopausal symptoms. Frequently reported symptoms of a menopausal syndrome include hot flashes, night sweats, menstrual irregularities, vaginal dryness, nervous tension, headaches, insomnia, and lack of energy [3, 37, 38], which are quite annoying. Previously we reported that ZRD could relieve some menopausal symptoms in mice. As expected, the independent activity time, grip strength time, sleeping time, thymus coefficient, and spleen coefficient were significantly decreased in ZRD treated mice compared with the young mice. On the contrary, the khan point and fat coefficients were significantly increased by ZRD [18]. These results implicated that ZRD could calm and ease the menopausal mice, inhibit the menopausal obesity, improve the immunity, and relieve fatigue. Besides, our new finding showed that ZRD could increase the weight of the uterus and reduce the weights of the adrenal gland in OVX rats (Figure 1). Moreover, ZRD could improve vaginal wall thickening and mucosa thickening and relieve atrophy, which was similar to TTL and EV groups as shown (Figure 3). Serum E2 levels were also elevated by ZRD (Figure 2(d)). These imply that ZRD might exert estrogenic effects through increasing the level of E2 and improving the uterine and vaginal tissue nutrition [32, 39]. Hence, ZRD might be a good choice for patients of genitourinary syndrome with bothersome symptoms such as vaginal dryness, itching, or dyspareunia [40].

Furthermore, ZRD showed excellent function in regulating the disturbance in hormones secretion. The onset of menopause is associated with a dramatic change in hormonal levels, a decrease in estrogen, and an increase in FSH and LH hormones, which causes permanent amenorrhea [41]. Under physiological condition, estrogen is mainly regulated by the hypothalamic-pituitary-ovarian axis (HPOA) [42]. Hypothalamic secretion of Luteinizing Hormone Releasing Hormone (LHRH) stimulates the pituitary secretion of FSH and LH, and FSH and LH promote the ovary secretion of estrogen (E2) [43]. Meanwhile, E2 regulates pituitary secretion of FSH and LH in negative feedback [42, 44]. The increased releasing of hormone LHRH results in increased secretion of FSH and LH, so the levels of FSH and LH in serum were higher in menopausal female rats [27]. Indeed, we found that ZRD could reduce the levels of FSH and LH in serum of OVX rats, while increasing E2 levels (Figure 2). Thus ZRD might relieve the disorders of menopausal syndrome by the HPOA.

In addition, the restoration of ER*α* and ER*β* expression might account for ZRD's protective effect [45]. Estrogen binds to estrogen receptors and exerts its impact on reproductive system [46, 47]. Meanwhile, ER*α* and ER*β* modulate the physiological functions of estrogenic compounds by regulating transcription of specific target genes. These two ERs share some common physiological roles, for example, in the development and function of the ovaries [48]. Here we found that the expressions of estrogen receptors were greatly decreased in OVX rats and ZRD could increase the expression of ER*α* and ER*β* both in the uterus and vagina (Figures 4 and 5). ER*α* is present mainly in uterus and ovary, showing a more prominent role in uterus [49]. Hence the increased vaginal epithelial thickness might result from increased ER*α* expression by ZRD. However, since it seems to have a more profound effect on the central nervous and immune systems [49, 50], the increased expression of ER*β* might account for the ZRD's protective effect in our previous report [18]. Consistently, recent report found that glycyrrhizae radix, cinnamomi cortex, Evodiae fructus, and Zingiberis rhizoma demonstrate ER*β*-dependent estrogenic activity and their combined use could produce synergistic ER*β*-dependent estrogenic activity [51]. In addition, the estrogen-stimulating bioactive proteins isolated from Dioscorea species could upregulate the protein expression of ER*β* and its translational levels, potentially reducing the risk of ovarian cancer [52]. These results scientifically support the traditional use of Chinese medicine for relieving menopausal syndrome or treating other female aging disorders, and progress in analysis techniques [53] would widely spread its potential clinical use in the future.

## 5. Conclusions

In summary, our present and previous study provide remarkable insight into the protective effect of ZRD on menopausal syndrome at molecular, morphological, and behavioral levels. Our work indicates that ZRD would be a new choice for menopausal patients. Next we would explore the constituents of ZRD and investigate their effects on expression of relevant genes and signaling pathways in future.

## Figures and Tables

**Table 1:** Compositions of ZhenRongDan.

**Chinese names**	**Latin name**	**English Names**	**Botanical Names**	**Part used**
熟地黄	RADIX REHMANNIAE PRAEPARATA	Prepared rehmannia root	Rehmannia glutinosa (Gaertn.) Libosch. ex Fisch. & C.A. Mey.	Dried root
白* 芍*	PAEONIAE RADIX ALBA	White peony root	Paeonia lactiflora Pall.	Dried root
*当 归*	ANGELICA SINENSIS RADIX	Chinese angelica	Angelica sinensis (Oliv.) Diels	Dried rhizome
*川 芎*	CHUANXIONG RHIZOMA	Ligusticum wallichii	Ligusticum chuanxiong S.H.Qiu, Y.Q.Zeng, K.Y.Pan, Y.C.Tang & J.M.Xu	Dried rhizome
牡丹皮	MOUTAN CORTEX	Moutan bark	Paeonia suffruticosa Andr.	Dried bark
丹参	SALVIA MILTIORRHIZAE RADIX ET RHIZOMA	Salvia miltiorrhiza	Salvia miltiorrhiza Bunge	Dried rhizome
鸡血藤	SPATHOLOBI CAULIS	Lignum millettiae	Spatholobus suberectus Dunn	Dried root
*肉苁蓉*	CISTANCHES HERBA	Desert living cistanche	Cistanche deserticola Y.C. Ma	Dried root
女贞子	LIGUSTRI LUCIDI FRUCTUS	Glossy privet fruit	Ligustrum lucidum W.T.Aiton	Dried fruit
*淫羊藿*	EPIMEDIUM FOLIUM	Short-horned Epimedium herb	Epimedium brevicornum Maxim.	Dried root
*续 断*	DIPSACI RADIX	Teasel root	Dipsacus asper Wall. Ex C.B. Clarke	Dried root
黄 柏	PHELLODENDRI CHINENSIS CORTEX	Golden cypress	Phellodendron chinense C.K.Schneid.	Dried bark
知 母	ANEMARRHENAE RHIZOMA	Common Anemarrhena rhizome	Anemarrhena asphodeloides Bunge	Dried rhizome
*炒酸枣仁*	ZIZIPHI SPINOSAE SEMEN	Parched spina date seed	Ziziphus jujuba Var. spinosa (Bunge) Hu ex H. F. Chow	Dried seed
山*茱萸*	CORNI FRUCTUS	Semen corni	Cornus officinalis Siebold & Zucc.	Dried fruit
*枸杞*	LYCII FRUCTUS	Chinese wolfberry	Lycium barbarum L.	Dried fruit
*甜菊甙*	STEVIA EXTRACT	Stevioside	Stevia rebaudiana (Bertoni) Bertoni	Leaf extract
